# Lianqiaoxinoside B, a Novel Caffeoyl Phenylethanoid Glycoside from *Forsythia suspensa*

**DOI:** 10.3390/molecules16075674

**Published:** 2011-07-04

**Authors:** Hai-Xue Kuang, Yong-Gang Xia, Jun Liang, Bing-You Yang, Qiu-Hong Wang

**Affiliations:** Key Laboratory of Chinese Materia Medica (Heilongjiang University of Chinese Medicine), Ministry of Education, Harbin 150040, China; Email: yonggangxia@163.com (Y.-G.X.); lliangjunn@163.com (J.L.); ybywater@163.com (B.-Y.Y.); qhwang668@sina.com (Q.-H.W.)

**Keywords:** *Forsythia suspensa*, caffeoylphenylethanoid glycoside, antioxidant and antimicrobial activities

## Abstract

Chemical investigation of the 70% ethanol extract of the unripe fruits of *Forsythia suspensa* resulted in the isolation of a novel caffeoyl phenylethanoid glycoside, lianqiaoxinoside B, together with the known compound forsythoside H. The new compound was elucidated to be 1'',2''-[*β-*(3,4,-dihydroxylphenyl)-*α*,*β*-dioxoethanol]-3''-*O*-caffeoyl-*O*-*α-*rhamnopyranosyl-(1→6)*-O*-*β-*glucopyranoside by extensive spectroscopic and chemical studies. Lianqiaoxinoside B and forsythoside H showed strong antioxidant and antimicrobial activities *in vitro* by the 2,2'-azinobis-3-ethylbenzothiazoline-6-sulphonate (ABTS) radical-scavenging assay and plate method. This study can be further extended to exploit for the possible application of caffeoyl phenylethanoid glycosides as the alternative antioxidants and antimicrobial agents of natural origin.

## 1. Introduction

**Figure 1:**
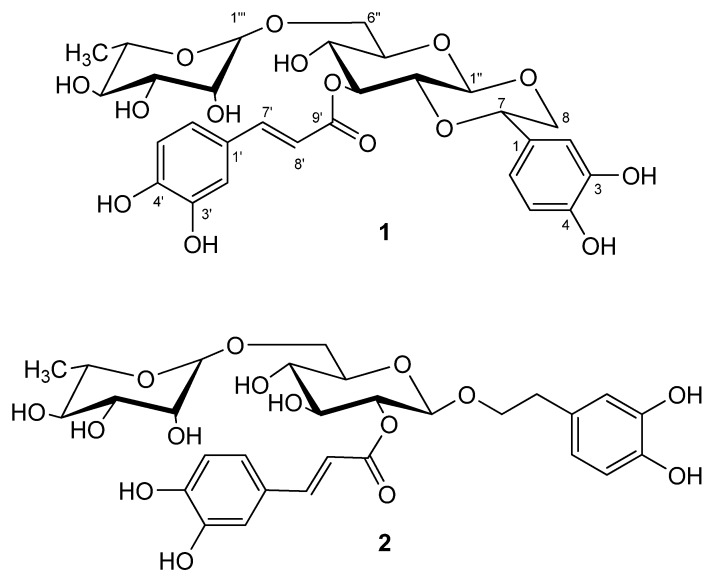
Structures of **1** and **2**.

*Forsythia suspensa* (Thunb.) Vahl, a small tree widely distributed in China, Korea, Japan and many European nations, belongs to the family Oleaceae [1]. The fruit of *F. suspensa* is also called in Chinese “Lianqiao” and has been used for antiinflammatory, diuretic, drainage and antidotal purposes [2]. The herb is listed in the Chinese Pharmacopoeia 2010 as an important crude drug and an aqueous extract is also used as a medicine. In previous papers, we reported the isolation of two phenylethanoid glycosides – forsythiaside and lianqiaoxinside A – and four lignans – phillygenin, (+)-isolariciresinol, phillyrin and (+)-pinoresinol-*β*-D-glucoside – from *F. suspensa* [2,3]. In this paper we report the isolation of lianqiaoxinoside B and forsythoside H from a 70% EtOH extract of unripe *F. suspensa* fruits. Their chemical structures (Figure 1) were elucidated by HRESIMS, 1D and 2D NMR techniques and chemical methods. The high antioxidant and antimicrobial activities of these two caffeoyl phenylethanoid glycosides were also examined.

## 2. Results and Discussion

### 2.1. Structure Elucidation of **1**

Compound **1** was obtained as a white amorphous powder and showed positive results for the *Molish* reagent. The UV spectrum of **1 **showed absorption band at 222, 246 (*sh*.), 287 (*sh*.) and 333 nm, respectively, which was considered to correspond to a caffeoyl phenylethanoid glycoside. Its molecular formula was established as C_2__9_H_3__4_O_1__5_ by the positive and negative HRESIMS from the [M+H]^+^, [M+NH_4_]^+^ and [M−H]^−^ signals at *m/z* 623.1996 (calc. for C_2__9_H_3__5_O_1__5_, 623.1976, [M+H]^+^), 640.2241 (calc. for C_2__9_H_38_NO_1__5_, 640.2241, [M+NH_4_]^+^), 621.1816 (calc. for C_2__9_H_3__3_O_1__5_, 621.1819, [M−H]^−^), respectively, indicating 13 degrees of unsaturation.

The ^1^H-NMR spectrum of **1 **exhibited several characteristic proton signals of the common caffeoyl phenylethanoid glycoside [δ 6.78(1H, *d*, *J = *1.2 Hz), 6.69(1H, *d*, *J = *8.2 Hz), and 6.66(1H, *dd*, *J = *8.2, 1.2 Hz); and 6.73(1H, *d*, *J = *8.2 Hz), 6.91(1H, *dd*, *J = *8.2, 2.0 Hz) and 7.01(1H, *d*, *J = *2.0 Hz)], a pair of *trans*-olefinic protons [δ 7.55 and 6.25 (each 1H, *d*, *J *= 16.0 Hz)]. In addition, two doublet signals due to anomeric protons at δ 4.51 (1H, *d*, *J *= 7.6 Hz), and 4.73 (1H, *d*, *J *= 0.8 Hz), suggested a diglycosidic structure for **1**. The glucose and rhamnose were further confirmed by ultra-performance liquid chromatography analysis based on pre-column derivatization with PMP reagent after acid hydrolysis of **1**. The configurations of the anomeric protons of glucose and rhamnose were proposed as *β* and *α*, respectively, on the basis of their coupling constants.

**Figure 2:**
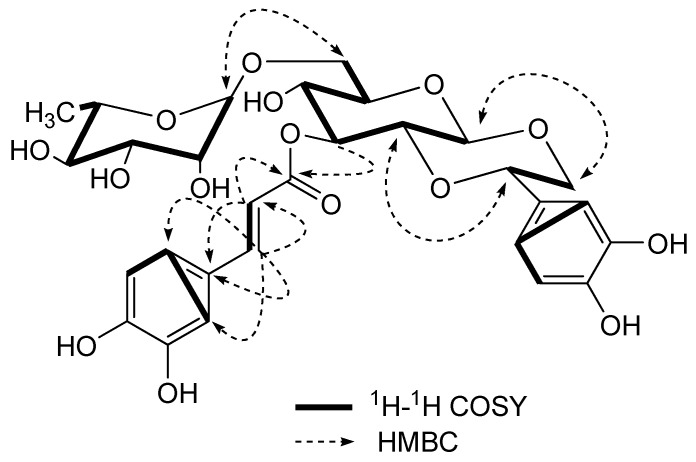
Key ^1^H-^1^H COSY and HMBC correlations of **1**.

^I3C-^NMR spectrum and DEPT experiments of **1 **showed 29 signals: one methyl, two methylenes, 19 methines and seven quaternary carbons, of which the signals at (δ 127.8, 115.5, 146.9, 149.8, 116.5, 123.1, 147.8, 114.6, 168.2) were attributed to the caffeoyl group. The caffeoyl site was established unambiguously by a HMBC experiment in which a long-range correlation between H-3′′ [δ 5.25(1H, *t*, *J = *8.8 Hz)] of the glucopyranosyl unit and the C-9′ (δ 168.2) of the caffeoyl residue was apparent (Figure 2). The rhamnopyranosyl site was established unambiguously by a HMBC experiment in which a long-range correlation between H-1′′′ [δ 4.73 (1H, *d*, *J = *0.8 Hz)] of the rhamnopyranosyl unit and the C-6′′ (δ 67.8) of the glucopyranosyl was apparent. Taking into account the NMR spectral data and the 13 degrees of unsaturation calculated from the empirical formula of **1**, it was suggested that **1** had another alicyclic ring except for an *α*, *β*-unsaturation carbonyl group, two aromatic rings, one rhamnosyl and one glucosyl. The linkage site of the additional ring was determined on the basis of the obvious HMBC correlations between H-1′′/C-8, H-8/C-1′′, H-2′′/C-7 and H-7/C-2′′, respectively. 

The relative stereochemistry of **1** was established by the coupling constants and NOESY correlations. The correlations of H-1'' with H-8a and H-3'', H-2'' with H-7 and H-4'' indicated that the glucopyranosyl and alicyclic rings all had a chair conformation with trans-fused ring junctions. In the ^1^H-NMR, the doublet at δ 4.51 (1H, *d*, *J = *7.6 Hz) suggested that the glucose was in the *β*-configuration, as well as the large coupling constant (*J* = 10.2 Hz) also indicated that H-7 and H-8a were similar to H-1' and H-2' in *trans*-axial orientation.

All the hydrogen and carbon signals of **1** have been fully assigned by combination of DEPT, ^1^H-^1^H COSY, HSQC, HMBC and NOESY experiments and comparison of spectral data with caffeoyl phenylethanoid glycosides reported in the literature [4]. The shift value for C-2 (δ 74.3) is found at a remarkably high field when compared with similar compounds [4], however, we considered that it is possibly due to the shielding effect of the caffeoyl group in the C-3 oxygen atom. The structure of **1** was thus assigned as 1'',2''-[*β*(3,4,-dihydroxylphenyl)-*α*,*β*-dioxoethanol]-3''-O-caffeoyl-O-*α-*rhamnopyranosyl-(1→6)*-*O-*β-*glucopyranoside, and was named lianqiaoxinoside B.

### 2.2. *In Vitro* Antimicrobial Activity

When studying the influence of the concentration of lianqiaoxinoside B and forsythoside H on the antimicrobial activities against bacterial strains we used two-fold microdilution broth method. The MIC data of the two compounds were presented in Table 1. The results showed both lianqiaoxinoside B and forsythoside H had high antimicrobial activities against the four common bacteria *B. vulgare*, *A. bacillus*, *M. pneumonia *and *B. dysenteriae*.

**Table 1:** Antimicrobial activity of different constituents of *F. suspensa *(MIC: μg/mL).

	1	2	Cefalexin
*S. aureus*	>200	>200	8.0
*E. coli*	>200	>200	4.0
*B. streptococci*	54.0	>200	8.0
*B. vulgare*	27.5	38.2	0.5
*A. bacillus*	31.5	36.5	0.5
*M. pneumoniae*	28.5	42.5	0.5
*S. albus*	>200	>200	8.0
*B. dysenteriae*	36.7	30.2	0.5

### 2.3. Antioxidant Activity

**Figure 3:**
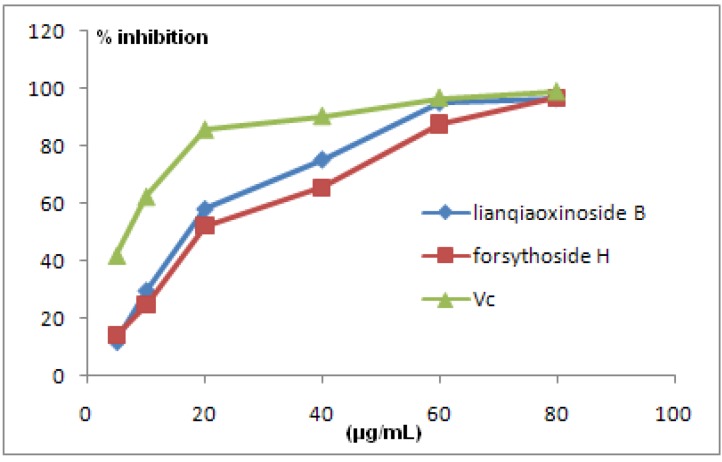
Scavenging effects of samples on ABTS radicals.

Recently, the significance of phenolic compounds as dietary antioxidants has been highlighted by experts [5,6,7]. The amount of phenol hydroxyls and amount of intramolecular H-bonding in a molecule play a key role in its antioxidant activity [5]. Many researchers have reported the strong antioxidant activity of rutin and chlorogenic acid. They are common natural phenolic antioxidants and they all have *ortho*-substituted hydroxyl structures [6]. The compounds lianqiaoxinoside B and forsythoside H exhibited significant ABTS radical scavenging ability, evidencing IC_50_ values of 15.6 and 17.7 μg/mL (Figure 3), compared with a positive control, Vc (IC_50_ 6.8 μg/mL). Recently many researches have demonstrated that forsythiaside, which is the marker and major constituent in this plant, possesses strong antioxidant activities [7]. Moreover, lianqiaoxinoside B and forsythoside H yielded nearly the same antioxidant activities. Obviously, all of these phenylethanoid glycosides have two *ortho*-substituting hydroxyl groups in both the caffeoyl and phenylethanoid moieties, which could be an important factor for their high antioxidant activity.

## 3. Experimental Section

### 3.1. General

IR spectra were recorded on a Shimadzu FTIR-8400S spectrometer. NMR spectra were recorded on a Bruker DPX 400 NMR instrument (400 MHz for ^1^H-NMR and 100 MHz for ^13^C-NMR). Chemical shifts are given as *δ *values with reference to tetramethylsilane (TMS) as internal standard, and coupling constants are given in Hz. HRESIMS were carried out on Waters Xevo QTOF mass spectrometer. Preparative HPLC (Waters, Delta 600–2487) was performed on a Hypersil-ODS II (10 m, 20 × 300 mm, Yilite, Dalian, People’s Republic of China).

### 3.2. Plant Material

The unripe fruits of *F. suspensa *were collected in March 2008 from Henan Province, China and identified by Prof. Wang Zhen-yue of Heilongjiang University of Chinese Medicine. The voucher specimen (2008009) was deposited at Herbarium of Heilongjiang University of Chinese Medicine, Harbin, China.

### 3.3. Extraction and Isolation

The unripe fruits of *F. suspensa *(10 kg) were ground and extracted under reflux conditions with 70% EtOH (30 L × 3 × 2 h each). The combined 70% EtOH extracts were evaporated to near dryness under vacuum and the resulting mixture (500 g) was suspended in H_2_O and partitioned successively with petroleum ether (3 × 2 L), CH_2_Cl_2_ (3 × 2.5 L), EtOAc (3 × 2.5 L) and *n*-BuOH (3 × 2.5 L). The *n*-BuOH fraction (90 g) was applied to a silica gel column chromatography with CH_2_Cl_2_/MeOH (15:1→1:1, *v/v*) gradient to give fractions B1→B7. Fraction B4 (18 g) was subjected to ODS column chromatography with MeOH/H_2_O (1:4→1:0, *v/v*) to yield ten sub-fractions C1→C10. Fraction C1 (300 mg) was purified by preparative HPLC on a Hypersil-ODS II column (10 μm, 20 mm × 300 mm, flow rate 8 mL·min^−1^) with MeOH/H_2_O (35:65) to afford **1** (10 mg, *t*_R_ = 12.6 min) and **2** (30 mg, *t*_R_ = 14.3 min). *Lianqiaoxinoside B* (**1**): White amorphous powder, [α] ^25^_D_ = +20.8 (c = 0.1, MeOH). IR (KBr): ν_max_ = 3399, 1698, 1607, 1523, 1282, 1117, 1055, 981, 813 cm^−1^. HRESIMS (positive): *m/z *= 623.1996 (calc. for C_29_H_35_O_15_, 623.1976, [M+H]^+^), 640.2241 (calc. for C_29_H_38_NO_15_, 640.2241, [M+NH_4_]^+^). HRESIMS (negative): *m/z *= 621.1816 (calc. for C_29_H_33_O_15_, 621.1819, [M−H]^−^). ^1^H- and ^13^C-NMR: see Table 2. *Forsythoside H* (**2**): White amorphous powder, [α]^ 25^_D_ = −15.7 (c = 0.1, MeOH). IR (KBr): ν_max_ = 3413, 1691, 1604, 1512, 1449, 1338, 1283, 1036 cm^−1^. HRESIMS (negative): *m/z *= 623.1954 (calc. for C_29_H_35_O_15_, 623.1976, [M−H]^−^). ^1^H- and ^13^C-NMR: see Table 2.

### 3.4. Determination of the *in-Vitro* Antimicrobial Activity

The experimental strains* Staphylococcus aureus*, *Escherichia coli*, *Beta-hemolytic streptococci*, *Bacterium vulgare*, *Aeruginosus bacillus*, *Micrococcus pneumoniae*, *S. albus and B. dysenteriae* were supplied by the Molecular Microbiology Laboratory of Heilongjiang University of Chinese Medicine. All the strains were tested for purity by Gram staining and by biochemical tests. The strains were kept at −70 °C in LB agar, activated by transferring into nutritive agar, and incubating at 37 ± 1.0 °C for 18 h. The antimicrobial activity against eight bacterial strains under different concentrations was determined by the plate method. A two-fold microdilution broth method was used to determinate the minimum inhibitory concentration (MIC) value. Details how to determine the antimicrobial activity were provided in a previously published paper [3].

**Table 2:** ^1^H- and ^13^C-NMR data of **1** and **2** in CD_3_OD at 400 MHz and 100 MHz, *J *in Hz.

No.	1			2	
	δ_H_	δ_C_		δ_H_	δ_C_
1		129.8			131.4
2	6.78 (1H, *d*, *J = *1.2 Hz)	115.1		6.59 (1H, *d*, *J = *1.6 Hz)	117.0
3		146.3			146.0
4		146.6			144.6
5	6.69 (1H, *d*, *J = *8.2 Hz)	116.4		6.57 (1H, *d*, *J = *8.0 Hz)	116.3
6	6.66 (1H, *dd*, *J = *8.2, 1.2 Hz)	119.5		6.48 (1H, *dd*, *J = *8.0, 1.6 Hz)	121.4
7	4.50 (1H, *dd*, *J = *10.2, 2.8 Hz)	78.7		2.67 (2H, *t*, *J = *7.2 Hz)	36.7
8	3.90 (1H, *dd*, *J = *12.0, 2.8 Hz)	72.6		3.94 (1H, *m*)	72.0
	3.61 (1H, *dd*, *J = *12.0, 10.2 Hz)			3.63 (1H, *m*)	
1′		127.8			127.8
2′	7.01 (1H, *d*, *J = *2.0 Hz)	115.5		7.06 (1H, *d*, *J = *1.2 Hz)	115.2
3′		146.9			146.8
4′		149.8			149.6
5′	6.73 (1H, *d*, *J = *8.2 Hz)	116.5		6.77 (1H, *d*, *J = *8.4 Hz)	116.5
6′	6.91 (1H, *dd*, *J = *8.2, 2.0 Hz)	123.1		6.91 (1H, *dd*, *J = *8.4, 1.2 Hz)	123.0
7′	6.25 (1H, *d*, *J = *16.0 Hz)	147.8		6.27 (1H, *d*, *J = *16.0 Hz)	147.1
8′	7.55 (1H, *d*, *J = *16.0 Hz)	114.6		7.56 (1H, *d*, *J = *16.0 Hz)	115.2
9′		168.2			168.4
1′′	4.51 (1H, *d*, *J = *7.6 Hz)	99.9		4.49 (1H, *d*, *J = *8.0 Hz)	102.4
2′′	3.40 (1H, *m*)	74.3		4.80 (1H, *t*, *J = *8.0 Hz)	75.1
3′′	5.25 (1H, *t*, *J = *8.8 Hz)	76.0		3.56 (1H, *t*, *J = *8.8 Hz)	76.2
4′′	3.62 (1H, *m*)	70.2		3.38 (1H, *m*)	71.7
5′′	3.67 (1H, *m*)	78.5		3.44 (1H, *m*)	76.9
6′′	3.45 (1H, *dd*, *J = *11.2, 5.2 Hz)	67.8		3.65 (1H, *dd*, *J = *10.8, 5.0 Hz)	67.9
	3.72 (1H, *dd*, *J = *11.2, 1.7 Hz)			4.00 (1H, *dd*, *J = *10.8, 1.2 Hz)	
1′′′	4.73 (1H, *d*, *J = *0.8 Hz)	102.3		4.74 (1H, *d*, *J = *1.6 Hz)	102.2
2′′′	3.85 (1H, *m*)	72.0		3.84 (1H, *m*)	72.3
3′′′	3.70 (1H, *m*)	72.4		3.70 (1H, *m*)	72.2
4′′′	3.37 (1H, *m*)	74.0		3.37 (1H, *m*)	74.0
5′′′	3.71 (1H, *m*)	69.9		3.70 (1H, *m*)	69.8
6′′′	1.26 (1H, *d*, *J = *6.5 Hz)	18.0		1.26 (1H, *d*, *J = *6.0 Hz)	18.1

### 3.5. ABTS Radical-Scavenging Assay

The radical scavenging capacity of antioxidant for the ABTS (2,2'-azinobis-3-ethylbenzothiazoline-6-sulphonate) radical action was determined as previously described [5,6,7]. ABTS was generated by mixing 7 mM of ABTS at pH 7.4 (5 mM NaH_2_PO_4_, 5 mM Na_2_HPO_4_ and 154 mM NaCl) with 2.5 mM potassium persulfate (final concentration) followed by storage in the dark at room temperature for 16 h before use. The mixture was diluted with ethanol to give an absorbance of 0.70 ± 0.02 units at 734 nm using spectrophotometer (Helios, Unicam, Cambridge UK). For each sample, the diluted methanol solution of essential oil (100 μL) was allowed to react with fresh ABTS solution (900 μL), and then the absorbance was measured 6 min after initial mixing. Ascorbic acid was used as a standard. The capacity of free radical scavenging was expressed by IC_50_ (mg/L) value, which represents the concentration required to scavenge 50% of ABTS radicals. The free radical scavenging activity of each solution was then calculated as percent inhibition according to the following equation: %inhibition = 100 (A (blank)-A (sample))/A (blank). 

### 3.6. Acid Hydrolysis and Derivatization with PMP Reagent

An aliquot of each sample (1.0 mg) was dissolved in 2M TFA (2 mL) in a 5 mL ampoule. The ampoule was sealed under a nitrogen atmosphere and kept in boiling water bath to hydrolyze samples into aglycone part and monosaccharides for 10 h. After the ampoule was cooled to room temperature, the reaction mixture was centrifuged at 3,000 rpm for 5 min. The supernatant was collected and methanol (1.5 mL) was added into it for the reaction mixture to be evaporated to dryness under a reduced pressure. Then the same amount of methanol was again added and dried by the same method as above, and the procedure was repeated thrice for TFA to be removed. The hydrolyzed and dried sample solutions were diluted with distilled water (2 mL) for the following experiments.

Two hundred μL of the hydrolyzed above sample was placed in 2.0 mL centrifuge tubes, then 0.5 M methanol solution (100 μL) of PMP and ammonia (200 μL) were added to each. Each mixture was allowed to react for 30 min in a 70 °C water bath, then cooled to room temperature and neutralized with formic acid (200 μL). The resulting solution was separated by liquid-liquid extraction using a volume of isoamyl acetate (two times) and chloroform (one time), respectively. After being shaken vigorously and centrifuged, the organic phase was carefully discarded to remove the excess reagents. Then the aqueous layer was filtered through a 0.22 μm membrane and diluted with water before UPLC analysis. The glucose and rhamnose were identified in **1** and **2** (rhamnose, *t*_R_ 2.03 min; glucose *t*_R_ 4.08 min). Details of the UPLC procedure can be seen our previous published paper [8].

## 4. Conclusions

Caffeoyl phenylethanoid glycosides are a category of important botanical ingredients which are extracted from many herb plants. As a part of our chemical investigation on the unripe fruits of* F. suspensa*, we have isolated a novel caffeoyl phenylethanoid glycoside with a 7,2′′-epoxy moiety and one known compound, forsythoside H. Their structures were established on the basis of spectroscopic and chemical evidence. It is also the first time the NMR data of forsythoside H in CD_3_OD is reported, which showed obvious difference with the spectrum in DMSO-*d*_6_ recorded in reference [9]. The pharmacological tests showed that they both exhibited high antioxidant and antimicrobial activities. This study can be further extended to exploit for the possible application of caffeoyl phenylethanoid glycosides as the alternative antioxidants and antimicrobial agents from natural origin. 
